# Establishment of a sheep immortalization cell line for generating and amplifying Orf virus recombinants

**DOI:** 10.3389/fvets.2022.1062908

**Published:** 2022-12-22

**Authors:** Shihui Sun, Kui Zhao, Huijun Lu, Xingyuan Liu, Yue Li, Qi Li, Deguang Song, Yungang Lan, Wenqi He, Feng Gao, Zi Li, Jiyu Guan

**Affiliations:** Key Laboratory of Zoonosis, Ministry of Education, College of Veterinary Medicine, Jilin University, Changchun, China

**Keywords:** immortalization cell line, *TERT*, OFTu, ORFV, ORFV recombinants

## Abstract

Orf virus (ORFV) causes highly contagious vesiculoulcerative pustular and skin lesions in ruminants like sheep. Developing ORFV-based recombinant vaccine is a potential way to combat Orf disease. Although ORFV could propagate in some kinds of primary cells, the proliferative capacity of primary cells is limited. Therefore, establishing immortalized stable cell line is an effective and affordable way for the production of live ORFV vaccine. In the present study, we introduced a telomerase reverse transcriptase (*TERT*) gene-expressing cassette into primary ovine fetal turbinate (OFTu) cells, then selected and expanded the cells, which was considered as immortalized OFTu cell line. Our results showed that *TERT* introduction has successfully expended the lifespan of OFTu cell line over 80 passages, without changing the cellular morphology, affecting chromosomes karyotype and inducing the cellular tumorigenic ability. Immortalized OFTu cell line-derived ORFV has caused similar levels of cytopathic effects (CPE), viral titers and viral particles when compared with the ORFV from primary OFTu cell. Importantly, immortalized OFTu cell line was suitable for generating gene-modified ORFV recombinant through homologous recombination, and for the amplification of ORFV recombinant. In summary, an immortalized OFTu cell line was established and characterized, which could be a powerful tool for preparing ORFV recombinant vaccines.

## Introduction

Orf virus (ORFV) belongs to the genus of *Parapoxvirus*, a member of *Poxviridae* family (1, 2). As a highly epitheliotropic virus, ORFV usually causes acute contact infection in goats, sheep and a variety of wild animals (1, 2). ORFV proliferates in the skin of lips, muzzle, nostrils, teats and oral mucosa (3). ORFV-caused lesions contain erythema, vesicles, pustules and scabs, which are usually limited to areas surrounding the viral infection sites and evolve resolved in 6 to 8 weeks. However, it has been reported that ORFV could harm herds through persistent infection (1, 2, 4). Of note, ORFV is also considered as a zoonotic virus, which could infect ranchers, shepherd and veterinarians (5). Due to ORFV-caused economic impact and public health issues, it is necessary to prevent and control Orf disease epidemic.

It was reported that establishing virulence gene-deletion ORFV recombinants is an effective way to develop potential vaccine (6). Several studies have established different ORFV recombinants with the deletion of different virulence genes, which may be the potential vaccines for combating Orf disease (7, 8). However, a cell-based platform is needed for the generation and production of gene-modified ORFV vaccine candidates.

Exploring suitable cells for viral production is also a key for the vaccine development (9). It has been reported that several primary cells were sensitive to ORFV infection. ORFV not only propagate in the epithelial and kidney cells of cattle and sheep, but also in testicular cells of calves and lambs. ORFV infection causes CPEs in these cells, while usually display relatively low viral titers (10). Other studies have shown that using primary nasal turbinate cells from fetal sheep for ORFV isolation has multiple advantages, including convenient culture, high efficiency for viral isolation, and high titers of proliferating ORFV (1, 2). However, primary ovine fetal turbinate (OFTu) cells have weak capacity of continuous proliferation, which limiting its application. More than that, considering the perspective of biosafety, the acquisition of these tissues mentioned above may carry the risk of Brucella infection. Cell lines are suitable for both clinical and basic research such as virus propagation and isolation. Therefore, it is important to establish an immortalized cell line for the production of ORFV, which could be an affordable and safe way for the production of live ORFV vaccine.

In this study, we tried to establish and characterize an immortalized OFTu cell line through *TERT* gene introduction and cell line evaluations. We further examined whether or not the immortalized OFTu cells are sensitive to ORFV infection. Furthermore, we also explored the capacity of immortalized OFTu cell line to generate and amplify gene-modified ORFV recombinants.

## Materials and methods

### Cells and viruses

Primary OFTu cells,Vero cells and HeLa cells were cultured in Dulbecco's Modified Eagle Medium (DMEM; Gibco) supplemented with 10% fetal bovine serum (FBS; Gibco), 100 U/mL penicillin and 100 mg/ml streptomycin. The WT ORFV strain OV-SY17 was isolated and characterized in our previous work (11). The OV-SY17-Δ120, in which the virulence gene *ORF120* was replaced with *EGFP*, was designed and modified as described in our previous work (8).

### Plasmid construction, transfection and selection

Human telomerase reverse transcriptase (*hTERT*) can activate telomerase activity, therefore maintains telomere length and extends cell lifespan (12, 13). The pCI-neo-*hTERT* was transfected into primary OFTu cells by Lipofectamine 3000 (#L3000015; ThermoFisher). 48 h after transfection, the cells were selected with G418 under the concentration of 300 μg/ mL. One week later, the concentration was reduced to 150 μg/ mL. Finally, we picked single cell clones and performed cell expansion.

### *HTERT* transcript level analysis

Total RNA was extracted from cells according to the RNAiso Plus protocols (9108; Takara), and First-strand cDNA was synthesized using the RNA to cDNA EcoDry Premix (639549; Clontech). The sequences of specific primers targeting *hTERT* were prepared as follows: forward, 5'-TCCGAGGTGTCCCTGAGTAT-3'; reverse, 5'-GACCCCAAAGAGTTTGCGAC-3'. The PCR conditions were as follows: 30 cycles of 94°C for 30s, 55°C for 30s and 72°C for 1 min using the TaKaRa Ex Taq^®^ (RR001A; Takara). The PCR products were loaded on 1% agarose gel along with a DL2000 DNA marker (3427A; Takara).

### Cell passage assay and cell viability assay

The continuous cell cultivation was performed with immortalized OFTu cells, and the passage ratio was gradually changed from 1:3 to 1:10. Each passage of cells was grown to a confluent monolayer, and the growth conditions of the passaged cells were recorded in Table 1.

**Table 1:** Immortalized OFTu cell line passage.

**Passage number**	**Passage ratio**	**Cell growth time (to a confluent monolayer, h)**
5	1:3	48.20 ± 0.50
10	1:3	48.25 ± 0.25
15	1:3	48.20 ± 0.52
20	1:4	52.30 ± 0.29
25	1:4	52.30 ± 0.50
30	1:4	52.20 ± 0.53
35	1:4	52.30 ± 0.29
40	1:5	56.20 ± 0.25
45	1:5	56.50 ± 0.29
50	1:5	56.27 ± 0.50
55	1:6	60.20 ± 0.53
60	1:6	60.50 ± 0.50
65	1:6	60.27 ± 0.29
70	1:10	72.50 ± 0.50
75	1:10	72.26 ± 0.53
80	1:10	72.50 ± 0.29

Data are expressed as mean ± S.D. (n = 3, each passage).

Cell viability assessed by CCK8 (HY-K0301; MCE) assay was performed according to the manufacturer's instructions, and detected the viability of immortalized OFTu cells (passage 15, 20) and primary cells (passage 15, 20).

### Chromosomal stability analysis

1 × 10^6^ cells were seeded in a 10 cm dish for 48 h, then incubated with 0.1 μg/mL colchicine (HY-16569; MCE) for 4 h. After trypsin digestion cells were treated with low osmotic pressure for 20 min at 37°C, and fixed by fixative solution (methanol: acetic acid =3:1). The fixed cells were dripped to the slide and stained by Giemsa, then observed by microscope.

### Xenograft assay

twelve female BALB/c nude mice were randomly divided into 4 groups and acclimatized for a week prior to the processing. The primary OFTu, immortalized OFTu and HeLa cells were hypodermically injected into the right dorsal of the mice at 1 × 10^7^ cells per mouse, and the same volume of PBS was injected as a blank control. The mice were monitored and measured once tumors were visible. The mice were sacrificed after 1 month.

All animal experiments in this study were conducted in strict accordance with the guidelines established by the Regulations on Requirements for Laboratory Animals and Laboratory Animal Environment and Housing Facilities of China and were approved by the College of Veterinary Medicine of Jilin University.

### Virus titration

Primary OFTu cells, immortalized OFTu cells and Vero cells were cultured in 96-well plates and incubated at 37°C and 5% CO_2_ to 70–80% confluency. Every column of 96-well plates was infected with 10-fold-dilution virus. Then the cells were observed daily for CPEs. The Karber's method was applied for calculating the TCID_50_ of this virus.

### Observation of viral particles

Primary OFTu cells and immortalized OFTu cells were infected with virus and harvested after 75% CPE, then applied three freeze-thaw cycles. In order to remove cellular debris, the viral supernatants were concentrated at 2000rpm for 20 min at 4°C. Then the viral supernatants were concentrated by ultracentrifuge (himac CP100WX) at 20000rpm for 2 h. Moreover, we performed density gradient centrifugation to obtain a high-purity virus solution. Finally, negative-stain transmission electron microscopy (TEM) was used to observing virions.

### Statistics

All animals were randomly grouped, and experiments were performed 2–3 times. Data are expressed as the means ± SD of the results of 3 separate experiments and analyzed by one-way analysis of variance (ANOVA) with uncorrelated Fisher's LSD multiple comparisons test with GraphPad Prism 8. Significance is presented as ^***^P < 0.001 and ^****^P < 0.0001. Other details of the statistical tests are described in the individual figure legends.

## Results

### Establishment and characterization of immortalized OFTu cell line

**Figure 1:**
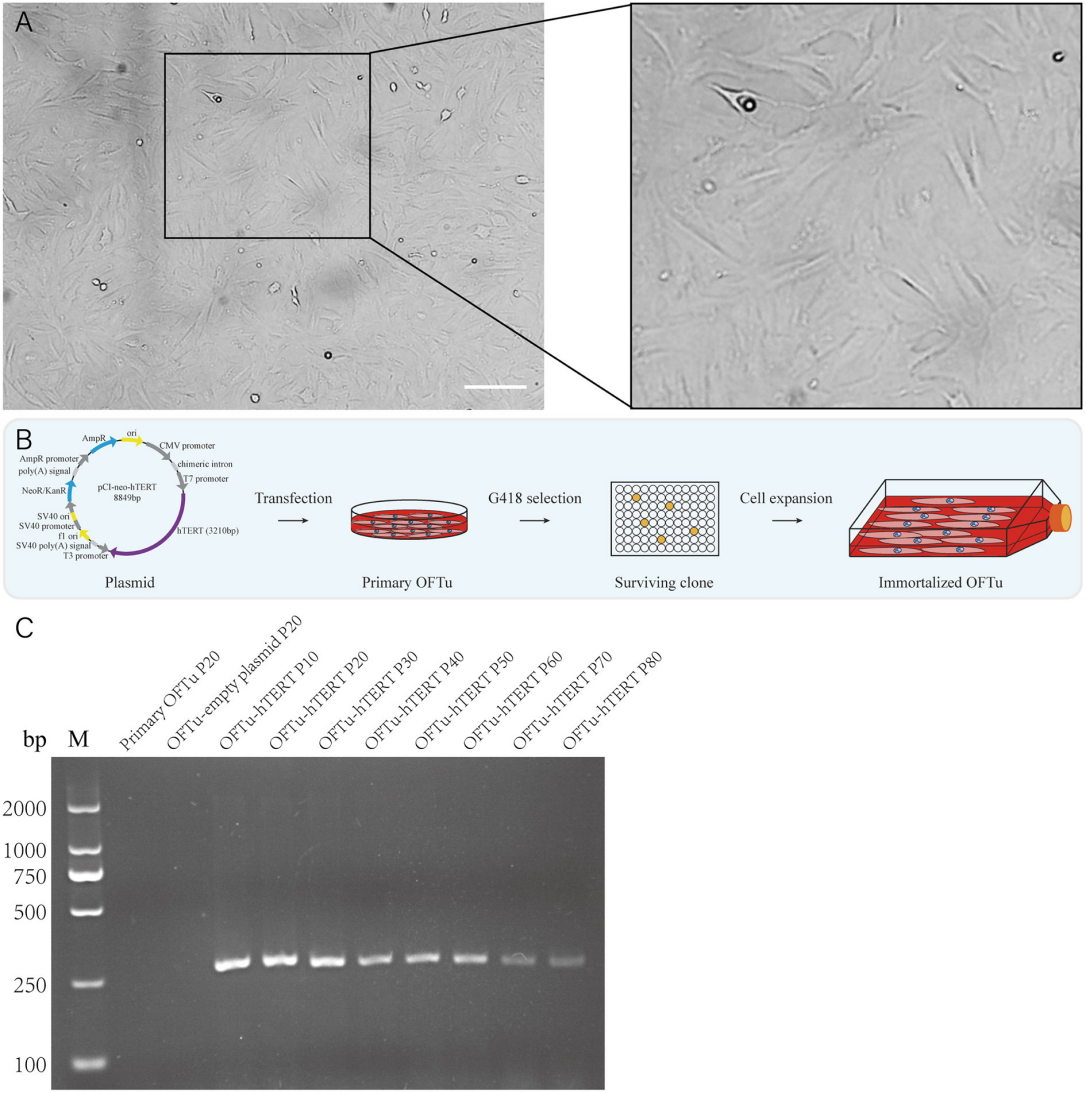
Generation of the OFTu cell line stably expressing *hTERT*. **(A)** Typical cell morphology of OFTu under phase contrast microscope. Scale bar = 100 μm. **(B)** Schematic overview to generate immortalized OFTu cell line expressing *hTERT*. **(C)** The *hTERT* gene transcripts were amplified from primary OFTu (lane 1), empty plasmid-transfected OFTu (lane 2) and *hTERT*-transfected OFTu (lane 3–10) by RT-PCR at several passages.

In order to establish immortalized OFTu cell line, we collected ovine fetal turbinate and further isolated the cells. The morphology of primary OFTu cells is shown in Figure 1A. These primary cells were passaged for 3–5 generations before the challenge with transgenes. Then, primary OFTu cells were transfected with *hTERT*-expressing plasmid, which was followed by the selection of geneticin sulfate (G418) until the formation of single cell clones (Figure 1B). Furthermore, one of the selected OFTu cell clones was passaged and *hTERT* transcription levels from different passages (10, 20, 30, 40, 50, 60, 70, and 80 passages) were examined. Compared with control groups (empty plasmid transfected and non-transfected cells), *hTERT* mRNA level can be detected in *hTERT*-transfected OFTu cells (Figure 1C). The above data demonstrate that we have obtained *hTERT* stably expressed OFTu cells.

**Figure 2:**
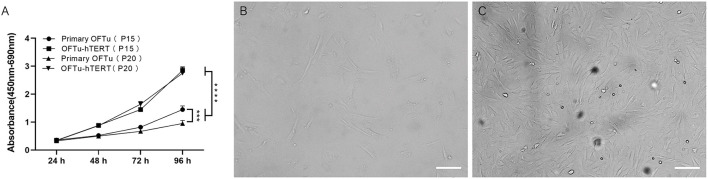
Cell morphology and growth characteristics of immortalized OFTu cells. **(A)** Immortalized OFTu at 15th and 20th passages showed a significantly stronger capacity for proliferation than primary OFTu at 15th and 20th passages. Primary OFTu passage 30 **(B)**, immortalized OFTu passage 80 **(C)** under phase contrast microscope. Scale bar = 100 μm. Data are expressed as the means ± SD of the results of 3 separate experiments and analyzed by one-way analysis of variance (ANOVA) with uncorrelated Fisher LSD multiple comparisons test with GraphPad Prism 8, ***P < 0.001, ****P < 0.0001.

We further performed cell viability assay and found *hTERT*-expressing cells from passage 15 and 20 grew significantly faster than corresponding primary OFTu cells (Figure 2A). While there was no significant difference between the *hTERT*-expressing OFTu cells from passage 15 and 20 (Figure 2A).

Next, the life span of *hTERT*-expressing cells by continuously passaging was recorded. We observed that non-transfected primary OFTu cells gradually stopped proliferating at around 30 passages (Figure 2B), while the *hTERT*-expressing cells could grow over 80 passages with normal cellular morphology (Figure 2C). The above data confirmed that immortalized OFTu cell line was established successfully. And compared with primary OFTu cells, there were no obvious cell morphological changes of *hTERT*-expressing cells under the observation of optical microscope (Figures 2B, C).

### Passaging characteristics of immortalized OFTu cell line

To determine the proliferative capacity of immortalized OFTu cells, continuous cell cultivation was performed. Interestingly, we were able to gradually change the passage ratio of immortalized OFTu cells from 1:3 to 1:10, while the cell growth time (to a confluent monolayer, h) was delayed from about 48 h to 72 h (Table 1). In contrast, the passaging ratio of primary OFTu cells was only 1:3 at most, otherwise the cells grew much slower and could not reach the expected confluence when the ratio was higher than 1:3 (data not shown).

### Immortalized OFTu is not available with tumorigenic ability

**Figure 3:**
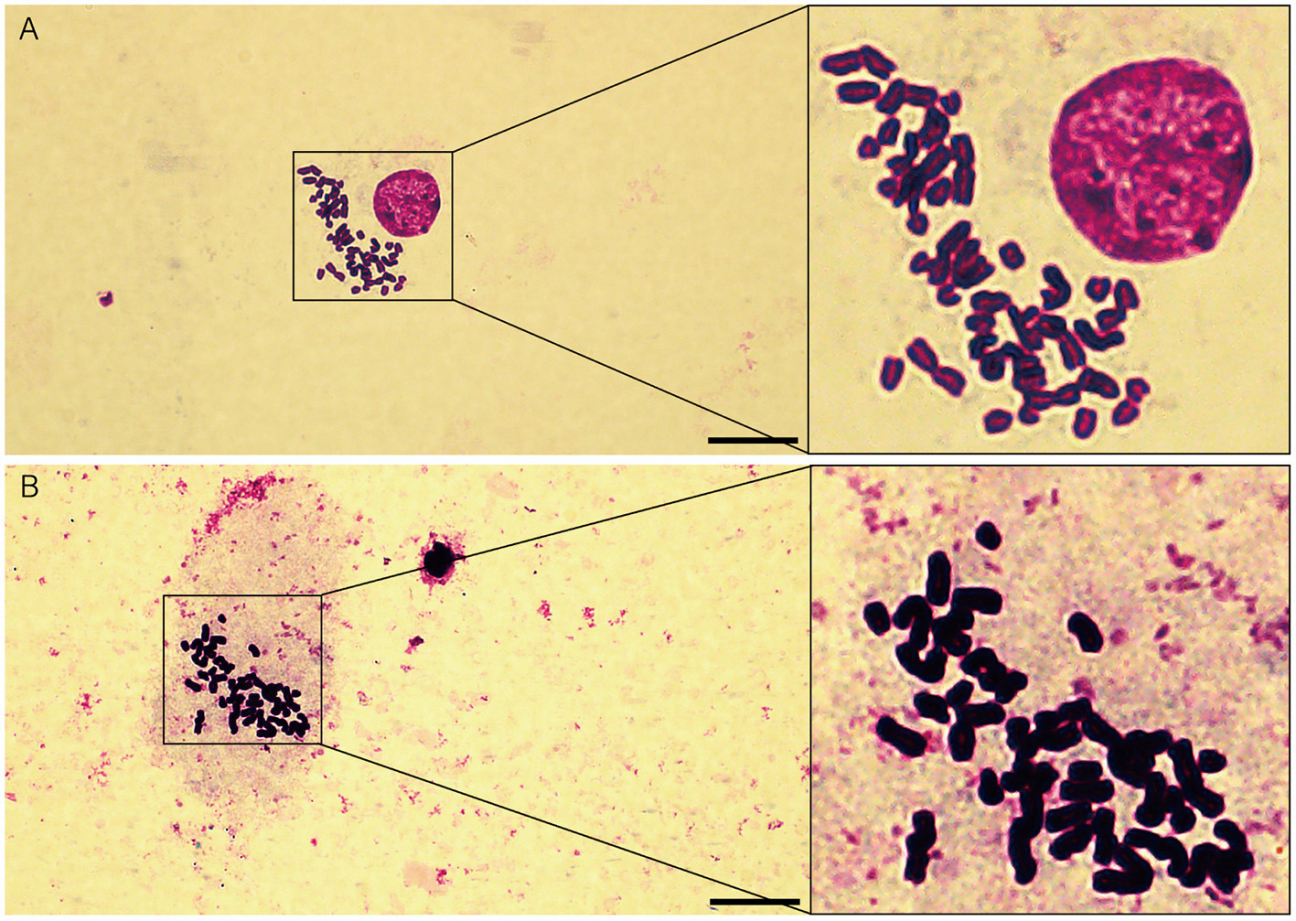
Chromosomes analysis of immortalized OFTu and primary OFTu. **(A)** Immortalized OFTu **(B)** Primary OFTu (passage 15). Scale bar = 20 μm.

In order to examine the chromosomes stability of immortalized OFTu cell line, karyotype analysis was performed. We found the immortalized OFTu cell line presented 27 pairs of chromosomes (Figure 3A), which was the same as those in primary OFTu cells (Figure 3B). The above data means that the chromosomes in immortalized OFTu cell line are stable.

**Figure 4:**
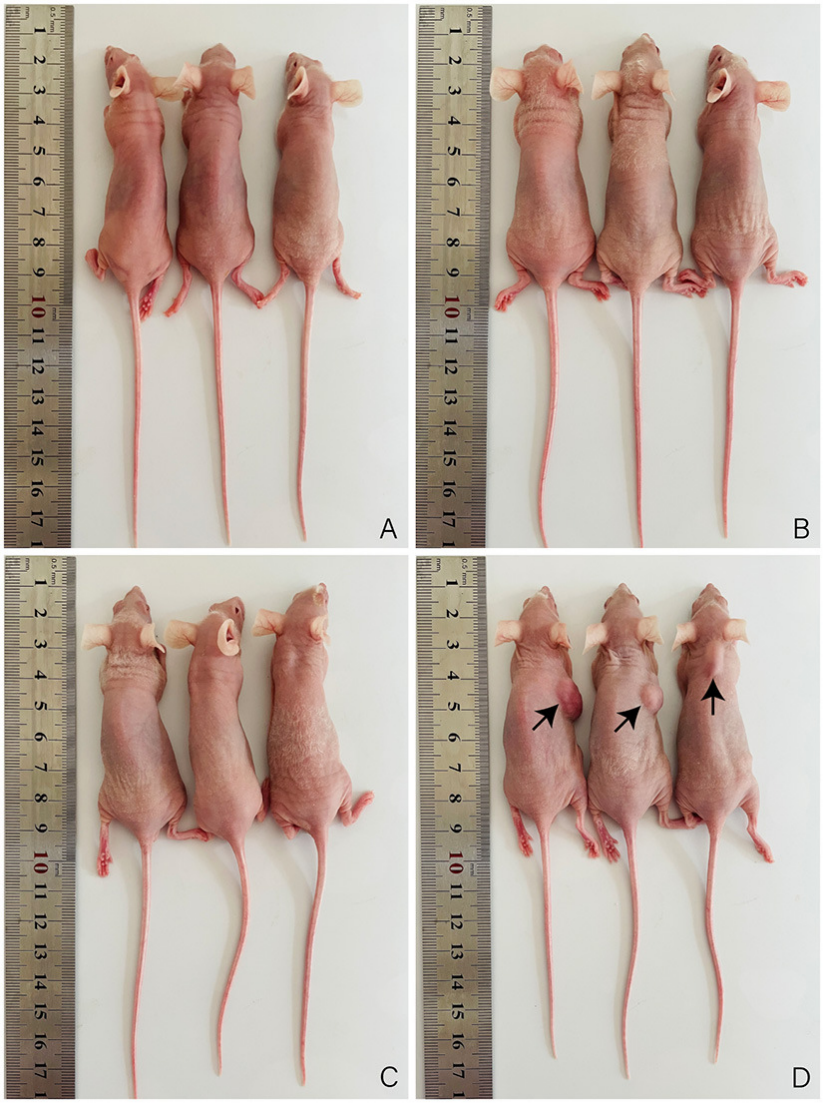
Immortalized OFTu cell line is not available with tumorigenic ability. Xenograft assay was performed to evaluate tumorigenic capacity of PBS **(A)** primary OFTu cells **(B)** immortalized OFTu cells **(C)**, and HeLa cells **(D)**.

To further examine whether immortalized OFTu cell has the ability of malignant transformation, we performed xenograft experiment by engrafting the immortalized OFTu cells into nude mice, and primary OFTu cell engraftment and PBS injection were performed as controls. Of note, Hela cells were also engrafted as the positive control. After cell engrafting, we have found that PBS group (Figure 4A), primary OFTu cell group (Figure 4B) and immortalized OFTu cell group (Figure 4C) were incapable of triggering the occurrence of tumors. In contract, mice inoculated with HeLa cells developed significant tumor masses (Figure 4D). Consequently, the immortalized OFTu cell line was not available with tumorigenic ability.

### ORFV propagates in immortalized OFTu cell line

**Figure 5:**
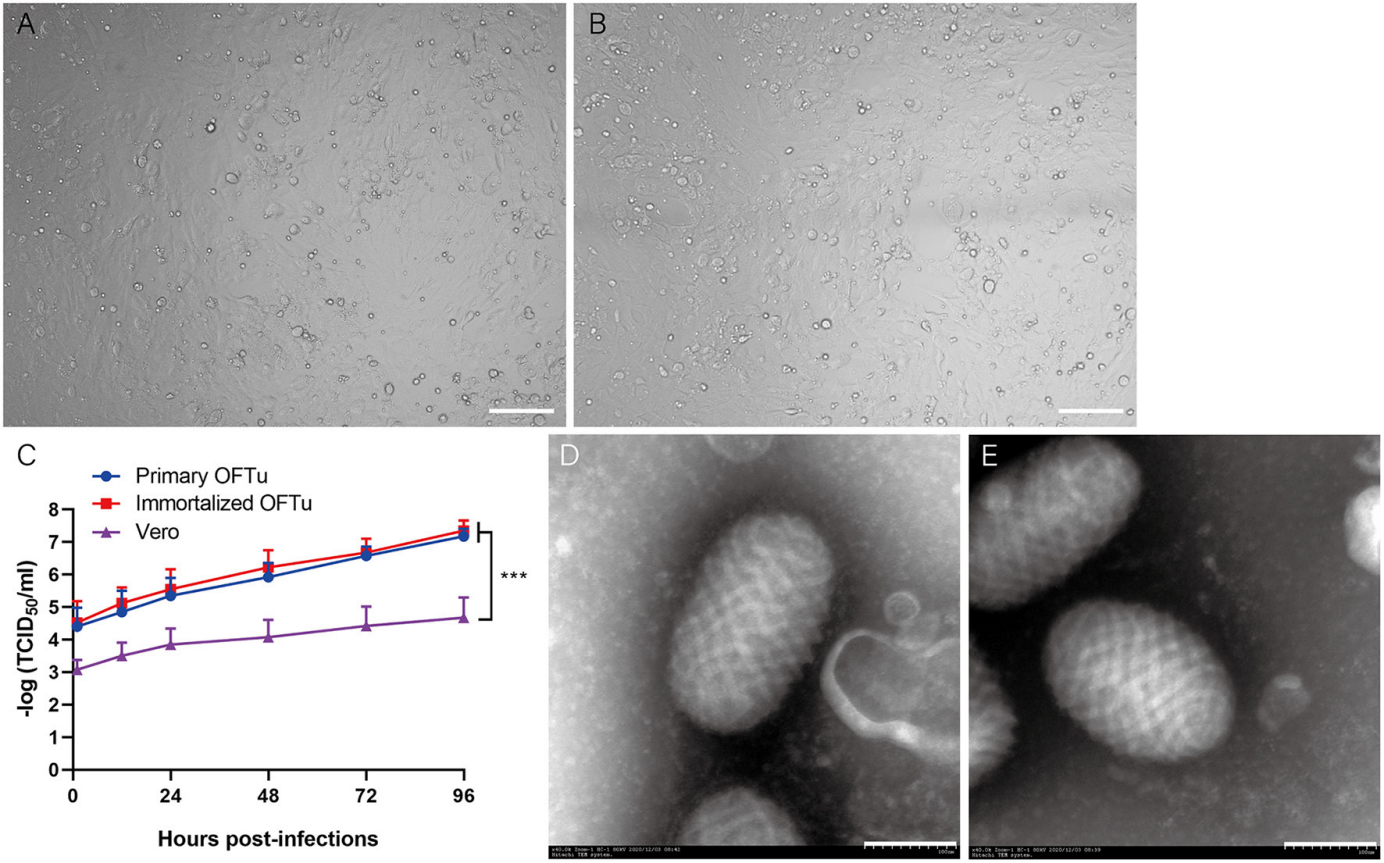
ORFV can propagates in immortalized OFTu cell line. CPE of immortalized OFTu **(A)** and primary OFTu cells **(B)** after ORFV infection (MOI = 1). Scale bar=100 μm **(C)** Titer of ORFV gathered from primary OFTu, immortalized OFTu and Vero cells. Data are expressed as the means ± SD of the results of 3 separate experiments and analyzed by one-way analysis of variance (ANOVA) with uncorrelated Fisher LSD multiple comparisons test with GraphPad Prism 8, ***P < 0.001. TEM observation of ORFV virions gathered from primary OFTu **(D)** and immortalized OFTu cells **(E)**. Scale bar = 100 nm.

It was reported that primary OFTu cells were sensitive to ORFV infection, which indicate that primary OFTu cell has the capacity to produce ORFV (1, 2). Here, we wonder whether or not immortalized OFTu was able to produce wild-type ORFV. To prove this, both immortalized and primary OFTu cells were infected with wild-type ORFV, which was followed by daily observations. Two days after ORFV infection, similar cytopathic effects (CPEs) were observed in the ORFV-infected immortalized and primary OFTu cells (Figures 5A, B). Subsequently, when 70–80 % OFTu cells underwent CPE, cell cultures were collected for TCID_50_ examination, with primary OFTu and Vero cells as controls. The results showed that there were no significant differences between immortalized and primary OFTu cells for ORFV production, as their TCID_50_ levels were almost the same (Figure 5C). While ORFV production from the above two kinds of cells were significantly more than that from Vero cells (Figure 5C). Furthermore, we collected all cell cultures and concentrated viral supernatants from immortalized and primary OFTu cells by ultracentrifuge, and then density gradient centrifugation was performed to obtain high-purity viruses. After observing viral particles through TEM, we found that viral particles from the immortalized OFTu cell line had similar size and morphology as those from primary OFTu cells (Figures 5D, E). The above data indicate that our established immortalized OFTu cell line is sensitive to ORFV and is able to produce high-quality ORFV particles.

### Immortalized OFTu cell line can generate and amplify gene-modified ORFV recombinants

**Figure 6:**
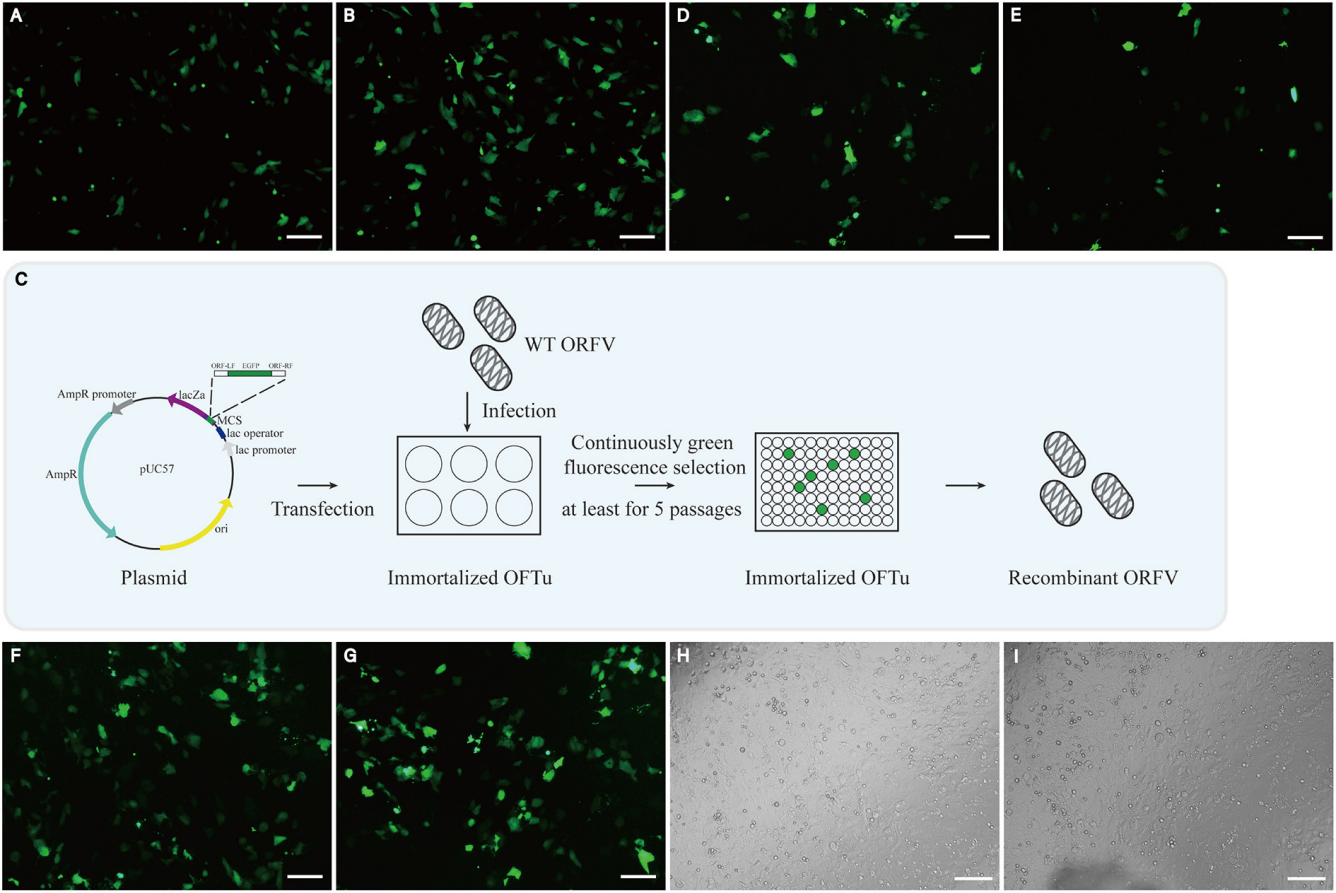
Immortalized OFTu cell line can generate and amplify recombinant ORFVs. Primary OFTu **(A)** and immortalized OFTu cell **(B)** under a fluorescence microscope 48 h after pEGFP-C3 transfection. Scale bar = 100 μm **(C)** Schematic overview to generate recombinant ORFV. Immortalized OFTu **(D)** and primary OFTu **(E)** produce GFP-positive recombinant viruses at 48 h.p.i. Immortalized OFTu **(F)** and primary OFTu **(G)** produce ORFV recombinant. Scale bar = 100 μm. Recombinant ORFV causes CPE in immortalized OFTu cells **(H)** and primary OFTu cells **(I)**. Scale bar = 100 μm.

Establishing gene-modified viruses is a core process for developing live recombinant vaccines. Therefore, we wonder whether or not immortalized OFTu cell line could be an appropriate platform for ORFV recombination. We firstly determined the transfection efficiency of immortalized OFTu cell line. pEGFP-C3 plasmid was transfected into the cells, which was followed by the observation of green fluorescence signal appearance. We found stronger green signal in plasmid-transfected immortalized OFTu cells than that in plasmid-transfected primary OFTu cells (Figures 6A, B). The above data indicates that the transfection efficiency is higher in immortalized OFTu cells, which provides a competitive advantage for the application of this cell line.

We have previously reported the generation of a kind of ORFV recombinant-OV-SY17-Δ120 in primary OFTu cell line. Briefly, a cassette containing an *EGFP* reporter and VV7.5 promoter has been used to replace *ORF120* gene in ORFV through homologous recombination, making the recombinant virus displaying green fluorescence in infected cells (8). Here, we wonder whether ORFV recombinant can also be generated in immortalized OFTu cell line. To prove this, we performed immortalized OFTu cell transfection, viral infection and virus recombinant selection as shown in the schematic illustration (Figure 6C). Interestingly, we found that it took around 48 h for the immortalized OFTu cell to generate GFP-positive recombinant viruses after ORFV infection (around 6 h after cassette transfection) (Figure 6D). In contrast, the quantity of GFP signal-positive primary cells was less than GFP signal-positive immortalized OFTu cells, which may be related to the difference in transfection efficiency (Figure 6E). After the generation of ORFV recombinant, we further infected the immortalized OFTu cell line with the viral recombinant, and found strong GFP signal in the cell line (Figure 6F). GFP signal with similar fluorescence intensity can also be found in ORFV recombinant-infected primary OFTu cells (Figure 6G). Next, we compared recombinant virus-caused CPE in the cells, and found that immortalized OFTu cell-derived GFP-positive recombinant virus was able to cause similar degree of CPE in both of these two kinds of cells (Figures 6H, I). The above data suggest the established immortalized OFTu cell line could be a suitable tool for generating and amplifying gene-modified ORFV recombinants.

## Discussion

In this study, to our knowledge, we describe for the first time an immortalized ovine fetal turbinate cell line. The immortalized OFTu cell line keeps the normal cellular morphology, while the lifespan reaches over 80 passages, which was much longer than that of the primary OFTu cells. We proved that immortalized OFTu cell line is non-tumorigenic, and could be a safe and effective tool for preparation of ORFV. Importantly, immortalized OFTu cell was capable for both generating and amplifying recombinant ORFV.

Normal cells cannot infinitely proliferate *in vitro*. After limited passages, they will gradually stop proliferating, getting senescent and eventually died. This phenomenon is called the Hefflick limit. Spontaneous cell immortalization was normally infrequent. Some cells can escape from the crisis of proliferation and senescence, and undergo spontaneously immortalization, like mouse mammary cells (14–16). In order to induce cells passing through unlimited proliferation, different kinds of methods have been explored. Carcinogenic agents (17), radiation (18) and even viral oncogenes (9, 19, 20) have been used to immortalize cells. However, carcinogenic agents like benzoapyrene (BaP) had tumorigenicity in nude mice, limiting its application (21). It was reported that radiation-induced immortalization occurred less frequently, caused cell morphological transformation and were tumorigenic (22, 23). As for oncogene-containing viruses such as EB virus (EBV), simian virus-40 (SV40) and human papillomavirus (HPV), they were reported to induce cell immortalization (19, 24, 25). But there is still a worry about that viral infection might not only change the phenotype of cells, but also transform them from benign into malignant. Here, we established immortalized OFTu cell line by activating telomerase, which is a commonly studied method and can add telomere on the end of chromosomes. Transfection with *hTERT* was used extensively for cell immortalization (13, 26–28). The activation or overexpression of *TERT* could be very effective in establishing cell line, avoiding causing tumorigenic and cell morphological transformation.

A recent study compared the sensitivity of three kinds of cells to ORFV (10). MDBK cell line was frequently used for preparing viruses, but the time of MDBK cells occurred CPE was much longer than other two kinds of cells after the infection with ORFV. Meanwhile, the virus titer in MDBK cells was relatively low, which was not suitable for ORFV propagation. Primary neonatal bovine testicular cells were also applied in ORFV culture and vaccine production (4), but the preparation of primary neonatal bovine testicular cells has biosafety risk. Although the titer of ORFV in bovine Sertoli cells was higher than the other two kinds of cells, its disadvantage was that it could not continuously cultivate over 16 passages (10). In contrast, it was reported that ORFV can propagate in OFTu cells (1, 2, 8, 29), and primary OFTu cells have been used in isolating ORFV from tissue samples (11). Compared with the other cells above, immortalized OFTu cell line with unlimited proliferative capacity and high sensitivity to ORFV was an ideal tool for ORFV production. Based on our data, although immortalized OFTu cell line has a much longer lifespan than primary OFTu cells, it still keeps the normal cellular morphology and proliferative capacity after continuous cultivation. Interestingly, when the passage ratio has been increased to 1:10, the cells still maintained high proliferative capacity (Table 1). Importantly, immortalized OFTu cells were more stable after gene transfection (Figure 6). This feature made immortalized OFTu cell line a valuable platform for generating gene-modified ORFV and producing them in a large scale.

## Conclusion

Here, we established an immortalized OFTu cell line by introducing an hTERT gene cassette, while did not confer tumorigenicity to the cell. This immortalized OFTu cell line could produce wild-type ORFV, and displayed capacity to generate and amplify gene-modified ORFV recombinant.

## Data availability statement

The raw data supporting the conclusions of this article will be made available by the authors, without undue reservation.

## Ethics statement

All animal experiments in this study were conducted in strict accordance with the guidelines established by the Regulations on Requirements for Laboratory Animals and Laboratory Animal Environment and Housing Facilities of China and were approved by the College of Veterinary Medicine of Jilin University.

## Author contributions

Conception and design: JG, SS, and KZ. Development of methodology: SS, HL, and KZ. Acquisition of data: SS, XL, YLi, and HL. Analysis and interpretation of data: SS, ZL, WH, DS, YLa, and FG. Writing review and revision of the manuscript: SS, JG, HL, and KZ. Administrative, technical, and material support: SS, FG, QL, ZL, WH, XL, and YLi. Study supervision: JG. All authors contributed to the article and approved the submitted version.

## Supplementary material

Supplementary Figure S1 The transfection efficiency is higher in immortalized OFTu cells.

Supplementary Figure S2 The quantity of GFP signal-positive in primary cells is less than GFP signal-positive immortalized OFTu cells.
