# Supplementary figures and images for: Establishment of a sheep immortalization cell line for generating and amplifying Orf virus recombinants
Source: Front Vet Sci. 2022 Dec 22;9:1062908. doi: 10.3389/fvets.2022.1062908 (PMC9813594; doi:10.3389/fvets.2022.1062908)

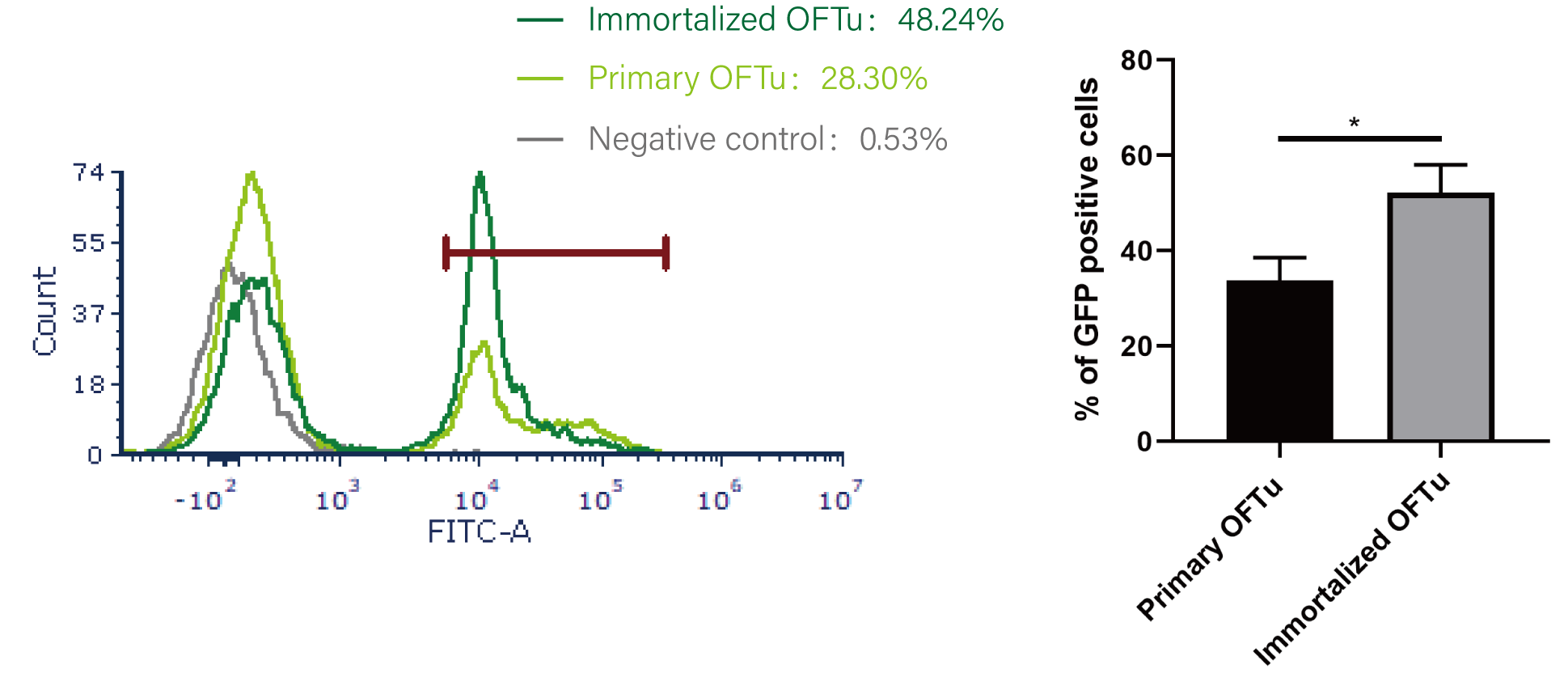

Supplement: Supplementary Figure S1 — The transfection efficiency is higher in immortalized OFTu cells. [file Image_1.TIF]

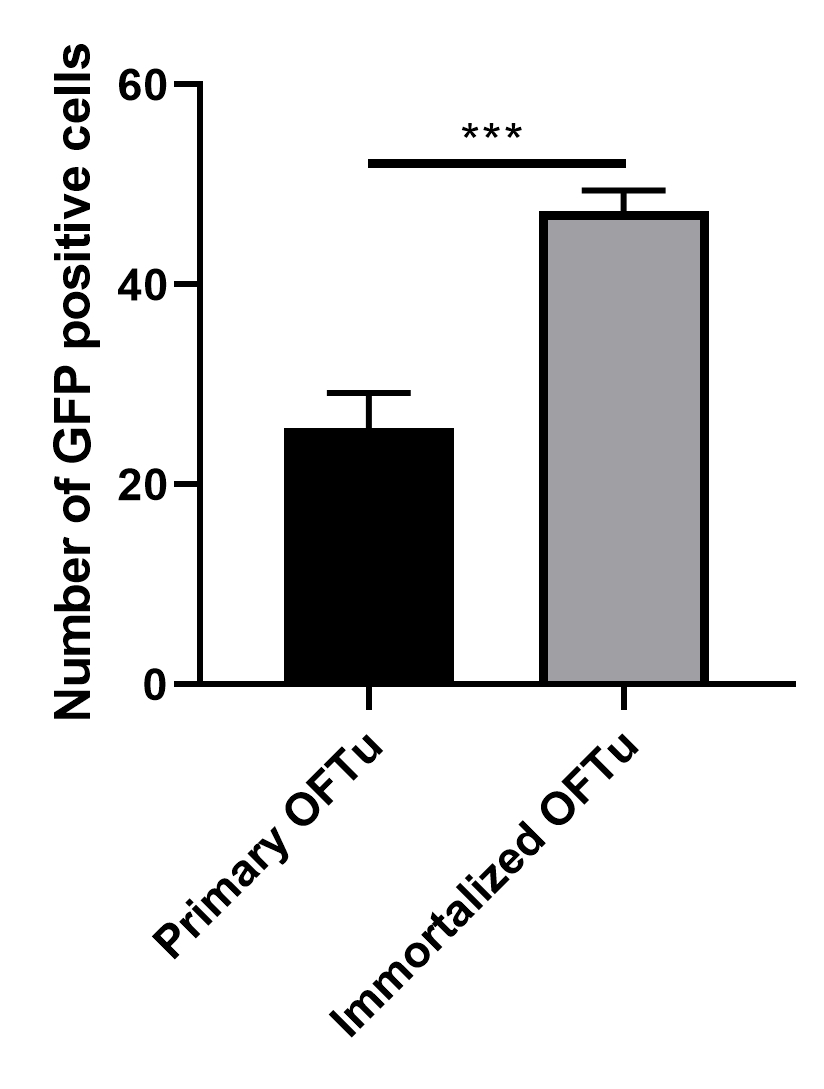

Supplement: Supplementary Figure S2 — The quantity of GFP signal-positive in primary cells is less than GFP signal-positive immortalized OFTu cells. [file Image_2.TIF]
